# Supplementary material for: Effects of biodegradable plastic film mulching on soil microbial communities in two agroecosystems
Source: PeerJ. 2020 Apr 20;8:e9015. doi: 10.7717/peerj.9015 (PMC7179572; doi:10.7717/peerj.9015)
Supplement: supplemental Information 1 [file peerj-08-9015-s001.docx]

**Supplemental Information**

**Table S1: Extracted DNA concentration and quality from soil samples collected from Spring 2015 to Fall 2016.**

| Treatment | Location | Time | DNA concentration (ng µl^-1^) | DNA quality (260/ ratio) |
| --- | --- | --- | --- | --- |
| BioAgri | TN | Fall 2015 | 13.3 | 2.45 |
| Naturecycle | TN | Fall 2015 | 18 | 1.87 |
| No Mulch | TN | Fall 2015 | 15.2 | 2.11 |
| Organix | TN | Fall 2015 | 16.5 | NA |
| PLA/PHA | TN | Fall 2015 | 10.4 | 2.14 |
| Polyethylene | TN | Fall 2015 | 10.6 | 1.95 |
| Weedguard | TN | Fall 2015 | 11.9 | 2.07 |
| BioAgri | TN | Fall 2016 | 10.2 | 1.83 |
| Naturecycle | TN | Fall 2016 | 12 | 1.94 |
| No Mulch | TN | Fall 2016 | 15.7 | NA*^a^* |
| Organix | TN | Fall 2016 | 14.6 | 1.91 |
| PLA/PHA | TN | Fall 2016 | 10.4 | 2 |
| Polyethylene | TN | Fall 2016 | 11.9 | 2.01 |
| Weedguard | TN | Fall 2016 | 11.4 | 1.95 |
| BioAgri | TN | Spring 2015 | 15.8 | 1.92 |
| Naturecycle | TN | Spring 2015 | 19.6 | 2.21 |
| No Mulch | TN | Spring 2015 | 11.8 | 2.17 |
| Organix | TN | Spring 2015 | 18 | NA |
| PLA/PHA | TN | Spring 2015 | 13.8 | 2.26 |
| Polyethylene | TN | Spring 2015 | 18.4 | 1.9 |
| Weedguard | TN | Spring 2015 | 14.9 | 1.93 |
| BioAgri | TN | Spring 2016 | 13.2 | 1.9 |
| Naturecycle | TN | Spring 2016 | 20.8 | 1.9 |
| No Mulch | TN | Spring 2016 | 19.6 | NA |
| Organix | TN | Spring 2016 | 19.7 | 1.9 |
| PLA/PHA | TN | Spring 2016 | 14.9 | 2.04 |
| Polyethylene | TN | Spring 2016 | 14.3 | 2.04 |
| Weedguard | TN | Spring 2016 | 14.9 | 1.92 |
| BioAgri | WA | Fall 2015 | 9.64 | 1.77 |
| Naturecycle | WA | Fall 2015 | 11.8 | 2.21 |
| No Mulch | WA | Fall 2015 | 14.4 | 2.01 |
| Organix | WA | Fall 2015 | 10.8 | NA |
| PLA/PHA | WA | Fall 2015 | 11.3 | 2.22 |
| Polyethylene | WA | Fall 2015 | 10.1 | 2.19 |
| Weedguard | WA | Fall 2015 | 14.3 | 2.21 |
| BioAgri | WA | Fall 2016 | 11.7 | 1.98 |
| Naturecycle | WA | Fall 2016 | 11.5 | 1.99 |
| No Mulch | WA | Fall 2016 | 13.7 | NA |
| Organix | WA | Fall 2016 | 11.7 | 1.88 |
| PLA/PHA | WA | Fall 2016 | 12.3 | 1.97 |
| Polyethylene | WA | Fall 2016 | 11 | 1.92 |
| Weedguard | WA | Fall 2016 | 10.4 | 1.99 |
| BioAgri | WA | Spring 2015 | 15.4 | 1.96 |
| Naturecycle | WA | Spring 2015 | 11.5 | 2.15 |
| No Mulch | WA | Spring 2015 | 13.7 | 2.07 |
| Organix | WA | Spring 2015 | 10.8 | NA |
| PLA/PHA | WA | Spring 2015 | 15.2 | 2.07 |
| Polyethylene | WA | Spring 2015 | 13.1 | 2.11 |
| Weedguard | WA | Spring 2015 | 13.4 | 2.14 |
| BioAgri | WA | Spring 2016 | 11.7 | 2.01 |
| Naturecycle | WA | Spring 2016 | 11.6 | 1.8 |
| No Mulch | WA | Spring 2016 | 11.2 | NA |
| Organix | WA | Spring 2016 | 11.2 | 1.86 |
| PLA/PHA | WA | Spring 2016 | 12.5 | 1.96 |
| Polyethylene | WA | Spring 2016 | 11.1 | 1.81 |
| Weedguard | WA | Spring 2016 | 14.9 | 1.95 |

*^a^*NA= data not collected.

**Table S2: The seven extracellular soil enzymes assayed for soils collected from Spring 2015-Spring 2017; their respective enzyme functions, substrates used for assays, and the role of each enzyme in biogeochemical cycling.** Labels used were MUB (4-methylumbelliferone) and MUC (7-amino-4-methylcoumarin).

| **Abbreviation** | **Enzyme name** | **Enzyme function** | **Substrate used** | **Indicator of microbial activity** |
| --- | --- | --- | --- | --- |
| XYL | β-xylosidase | hemicellulose degradation | 4-MUB-β-D- xylopyranoside | Carbon cycling |
| BG | β-glucosidase | sugar degradation | 4-MUB-β-D-glucopyranoside | Carbon cycling |
| AG | α- glucosidase | sugar degradation | 4-MUB-α-D-glucopyranoside | Carbon cycling |
| NAG | N-acetyl β glucosaminidase | chitin degradation | 4-MUB-N-acetyl- | Carbon and Nitrogen cycling |
|  |  | β-D-glucosaminide |  |  |
| CB | β-D cellubiosidase | cellulose degradation | 4-MUB-β-D-cellobioside | Carbon cycling |
| PHOS | Phosphatase | phosphorus mineralization | 4-MUB phosphate | Phosphorus cycling |
| LAP | Leucine amino peptidase | protein degradation | L- leucine-7-amido-4-methylcoumarin hydrochloride | Nitrogen mineralization |

**Table S3: Weather data at ETREC, Knoxville, TN and NWREC, Mount Vernon, WA.** Adapted from Sintim et al. (2019).

| Year*^a^* | Average relative humidity (%) | Average wind speed (ms^-1^) | Air temperature  T_min_ (ºC) T_mean_ (ºC) T_max_ (ºC) | | | Total precipitation (mm) |
| --- | --- | --- | --- | --- | --- | --- |
| Knoxville (January to December) | | | | | | |
| 2015 | NA*^b^* | 1.24 | 10.4 | 15.8 | 21.2 | 1313 |
| 2016 | 74.1 | 1.55 | 10.0 | 15.8 | 22.6 | 866 |
| 2017 | 76.9 | 1.56 | 10.3 | 15.7 | 22.0 | 1374 |
| Mount Vernon (January to December) | | | | | | |
| 2015 | 82.7 | 1.65 | 6.97 | 11.6 | 16.6 | 885 |
| 2016 | 79.7 | 1.81 | 7.32 | 11.5 | 16.1 | 945 |
| 2017 | 80.1 | 1.70 | 5.95 | 10.4 | 15.3 | 987 |

*^a^*In Knoxville, 2015 data was taken from National Oceanic and Atmosphere Administration (NOAA) station at Knoxville McGhee Tyson Airport, 12 km from the field site, and the 2016 and 2017 data were taken from a weather station installed at the research site. In Mount Vernon, the 2015, 2016, and 2017 data were taken from Washington State University AgWeatherNet Station located 100 m from the field site. T_min_, T_mean_, T_max_: minimum, mean, and maximum air temperature, respectively. *^b^*NA = not available.

**Table S4: PERMANOVA results for comparisons between microbial community compositions.** Table generated using adonis function (vegan package) in R statistical environment. Df: degrees of freedom, Sums of Sqs: sequential sums of squares, Mean Sqs: mean squares F.model: F statistics, R^2^: Partial R squared.

|  | **Df** | | **Sums of Sqs** | | **Mean Sqs** | | **F.Model** | | **R^2^** | | **P value** | |  |
| --- | --- | --- | --- | --- | --- | --- | --- | --- | --- | --- | --- | --- | --- |
| *Location* |  | |  | |  | |  | |  | |  | |  |
| Location | 1 | | 1.228 | | 1.228 | | 117.34 | | 0.414 | | 0.001 *** | |  |
| Residuals | 166 | | 1.737 | | 0.01 | |  | | 0.586 | |  | |  |
| Total | 167 | | 2.966 | |  | |  | | 1 | |  | |  |
| *Seasons in TN* |  | |  | |  | |  | |  | |  | |  |
| Season_Year | 3 | | 0.436 | | 0.145 | | 17.831 | | 0.401 | | 0.001 *** | |  |
| Residuals | 80 | | 0.651 | | 0.008 | |  | | 0.599 | |  | |  |
| Total | 83 | | 1.087 | |  | |  | | 1 | |  | |  |
| *Treatments (TN)* |  | |  | |  | |  | |  | |  | |  |
| *Spring 2015* |  | |  | |  | |  | |  | |  | |  |
| Treatment | 6 | | 0.027 | | 0.004 | | 0.61 | | 0.207 | | 0.893 | |  |
| Residuals | 14 | | 0.103 | | 0.007 | |  | | 0.793 | |  | |  |
| Total | 20 | | 0.13 | |  | |  | | 1 | |  | |  |
| *Fall 2015* |  | |  | |  | |  | |  | |  | |  |
| Treatment | 6 | | 0.046 | | 0.008 | | 0.865 | | 0.27 | | 0.598 | |  |
| Residuals | 14 | | 0.125 | | 0.009 | |  | | 0.73 | |  | |  |
| Total | 20 | | 0.171 | |  | |  | | 1 | |  | |  |
| *Spring 2016* |  | |  | |  | |  | |  | |  | |  |
| Treatment | 6 | | 0.034 | | 0.006 | | 0.843 | | 0.265 | | 0.607 | |  |
| Residuals | 14 | | 0.094 | | 0.007 | |  | | 0.735 | |  | |  |
| Total | 20 | | 0.128 | |  | |  | | 1 | |  | |  |
| *Fall 2016* |  | |  | |  | |  | |  | |  | |  |
| Treatment | 6 | | 0.073 | | 0.012 | | 1.146 | | 0.329 | | 0.313 | |  |
| Residuals | 14 | | 0.149 | | 0.011 | |  | | 0.671 | |  | |  |
| Total | 20 | | 0.223 | |  | |  | | 1 | |  | |  |
| *Seasons in WA* |  | |  | |  | |  | |  | |  | |  |
| Season_Year | 3 | | 0.359 | | 0.12 | | 32.835 | | 0.552 | | 0.001 *** | |  |
| Residuals | 80 | | 0.292 | | 0.004 | |  | | 0.448 | |  | |  |
| Total | 83 | | 0.65 | |  | |  | | 1 | |  | |  |
| *Treatments (WA)* | |  | |  | |  | |  | |  | |  | |
| *Spring 2015* | |  | |  | |  | |  | |  | |  | |
| Treatment | | 6 | | 0.013 | | 0.002 | | 0.806 | | 0.257 | | 0.86 | |
| Residuals | | 14 | | 0.037 | | 0.003 | |  | | 0.743 | |  | |
| Total | | 20 | | 0.05 | |  | |  | | 1 | |  | |
| *Fall 2015* | |  | |  | |  | |  | |  | |  | |
| Treatment | | 6 | | 0.039 | | 0.006 | | 1.964 | | 0.457 | | 0.004 ** | |
| Residuals | | 14 | | 0.046 | | 0.003 | |  | | 0.543 | |  | |
| Total | | 20 | | 0.085 | |  | |  | | 1 | |  | |
| *Spring 2016* | |  | |  | |  | |  | |  | |  | |
| Treatment | | 6 | | 0.011 | | 0.002 | | 0.805 | | 0.257 | | 0.887 | |
| Residuals | | 14 | | 0.033 | | 0.002 | |  | | 0.743 | |  | |
| Total | | 20 | | 0.044 | |  | |  | | 1 | |  | |
| *Fall 2016* | |  | |  | |  | |  | |  | |  | |
| Treatment | | 6 | | 0.04 | | 0.007 | | 1.259 | | 0.35 | | 0.167 | |
| Residuals | | 14 | | 0.073 | | 0.005 | |  | | 0.65 | |  | |
| Total | | 20 | | 0.113 | |  | |  | | 1 | |  | |
|  | |  | |  | |  | |  | |  | |  | |
|  | |  | |  | |  | |  | |  | |  | |

**Table S5: Mean richness (number of unique OTUs) and diversity (inverse Simpson index) estimates for bacterial communities by location, season and treatment.**

| Time | Treatment | **TN** | | **WA** | |
| --- | --- | --- | --- | --- | --- |
|  |  | Number of unique OTUs  (mean±SEM) | Inverse Simpson index  (mean±SEM) | Number of unique OTUs  (mean±SEM) | Inverse Simpson index  (mean±SEM) |
| Spring 2015 | BioAgri | 282 ± 5 | 8.000 ± 0.405 | 272 ± 4 | 8.244 ± 0.214 |
|  | Naturecycle | 269 ± 5 | 7.723 ± 0.36 | 273 ± 3 | 7.938 ± 0.108 |
|  | Organix | 277 ± 5 | 7.409 ± 0.281 | 270 ± 2 | 8.153 ±0.114 |
|  | PLA/PHA | 266 ± 4 | 8.2 ± 0.89 | 273 ± 6 | 8.345 ± 0.166 |
|  | Weedguard | 273 ± 3 | 8.012 ± 0.234 | 275 ± 7 | 8.098 ± 0.381 |
|  | Polyethylene | 283 ± 8 | 7.679 ± 0.525 | 278 ± 2 | 8.391 ± 0.457 |
|  | No mulch | 268 ± 7 | 7.634 ± 0.131 | 274 ± 3 | 8.402 ± 0.55 |
| Fall 2015 | BioAgri | 280 ± 14 | 7.918 ± 0.259 | 261 ± 5 | 9.095 ± 0.369 |
|  | Naturecycle | 281 ± 10 | 8.031 ± 0.406 | 269 ± 2 | 9.031 ± 0.281 |
|  | Organix | 271 ± 6 | 7.685 ± 0.232 | 259 ± 4 | 8.804 ± 0.143 |
|  | PLA/PHA | 268 ± 7 | 8.255 ± 0.444 | 250 ± 4 | 8.053 ± 0.085 |
|  | Weedguard | 272 ± 4 | 8.199 ± 0.114 | 264 ± 11 | 8.43 ± 0.318 |
|  | Polyethylene | 254 ± 1 | 7.599 ± 0.61 | 252 ± 3 | 8.825 ± 0.217 |
|  | No mulch | 293 ± 7 | 7.792 ± 0.248 | 280 ± 2 | 9.926 ± 0.796 |
| Spring 2016 | BioAgri | 303 ± 6 | 8.195 ± 0.22 | 270 ± 9 | 8.254 ± 0.305 |
|  | Naturecycle | 284 ± 7 | 8.216 ± 0.379 | 271 ± 8 | 8.027 ± 0.118 |
|  | Organix | 292 ± 8 | 7.875 ± 0.411 | 272 ± 3 | 8.028 ± 0.143 |
|  | PLA/PHA | 286 ± 2 | 8.341 ± 0.173 | 269 ± 9 | 8.196 ± 0.215 |
|  | Weedguard | 284 ± 6 | 8.579 ± 0.206 | 270 ± 6 | 8.01 ± 0.227 |
|  | Polyethylene | 281 ± 5 | 7.333 ± 0.381 | 270 ± 4 | 8.394 ± 0.169 |
|  | No mulch | 287 ± 10 | 7.713 ± 0.22 | 278 ± 5 | 8.302 ± 0.255 |
| Fall 2016 | BioAgri | 299 ± 8 | 10.18 ± 0.463 | 272 ± 3 | 10.32 ± 0.397 |
|  | Naturecycle | 294 ± 3 | 10.53 ± 0.302 | 278 ± 4 | 9.525 ± 0.615 |
|  | Organix | 288 ± 4 | 9.234 ± 0.27 | 275 ± 6 | 9.479 ± 0.867 |
|  | PLA/PHA | 284 ± 2 | 10.32 ± 0.77 | 277 ± 5 | 10.31 ± 0.76 |
|  | Weedguard | 288 ± 10 | 11 ± 0.961 | 277 ± 2 | 11.45 ± 0.822 |
|  | Polyethylene | 271 ± 6 | 8.777 ± 0.436 | 275 ± 4 | 10.65 ± 0.677 |
|  | No mulch | 290 ± 7 | 9.967 ± 0.312 | 277 ± 7 | 10.12 ± 0.382 |

**Table S6: T values from paired t-tests comparing 16S rRNA and ITS gene copy initial abundances (Spring 2015) to final abundances (Fall 2016) to determine significant changes over the two-year experiment in Knoxville, TN and Mount Vernon, WA.** Significant values are in bold, *p < 0.05; **p < 0.01; ***p < 0.001.

| **Mulch Treatments and Controls** | | **TN** | | **WA** | |
| --- | --- | --- | --- | --- | --- |
|  |  |  |  |  |  |
|  |  | 16S | ITS | 16S | ITS |
|  | BioAgri | 0.83 | 0.76 | **-4.30*** | -2.27 |
|  |  |  |  |  |  |
| Treatments | Naturecycle | -1.30 | -1.45 | -4.02 | **-6.87*** |
|  | Organix | **-3.9*** | -0.52 | **-4.30*** | -3.05 |
|  | Experimental PLA/PHA | **-3.51*** | -0.20 | **-8.34**** | **-5.64**** |
|  | Weedguard | -0.80 | -0.89 | **-3.52*** | -1.50 |
| Controls | Polyethylene | **-4.06*** | -0.23 | -2.53 | -0.02 |
|  | No mulch | -0.65 | -1.21 | -1.74 | -0.38 |


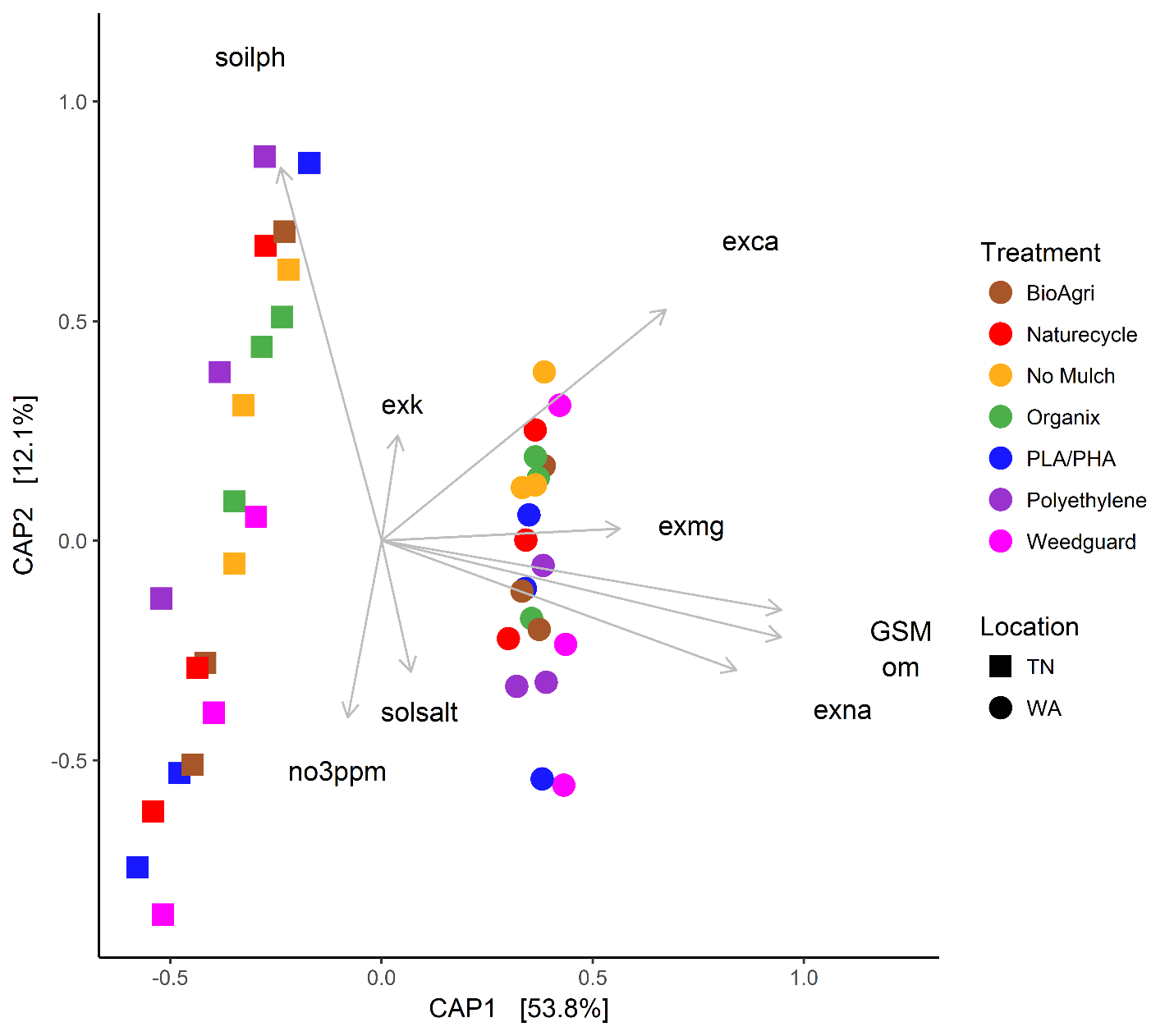


**Fig. S1: Canonical analysis of principal coordinates (CAP) of soil communities collected in Fall 2016 season.** Ordination constrained by environmental variables: soilph: soil pH, solsalt: electrical conductivity (dS/m), exk: exchangeable potassium by NH_4_OAc extraction (mg/ kg), exmg: exchangeable magnesium by NH_4_OAc extraction (mg/ kg), exca: exchangeable calcium by NH_4_OAc extraction (mg/ kg), exna: exchangeable sodium by NH_4_OAc extraction (mg/ kg), no3ppm: Nitrate-N (mg/kg), GSM: Gravimetric soil moisture (g water/ g dry soil), om: Organic matter (%). Data for environmental variables are reported in Sintim et al. (2019).


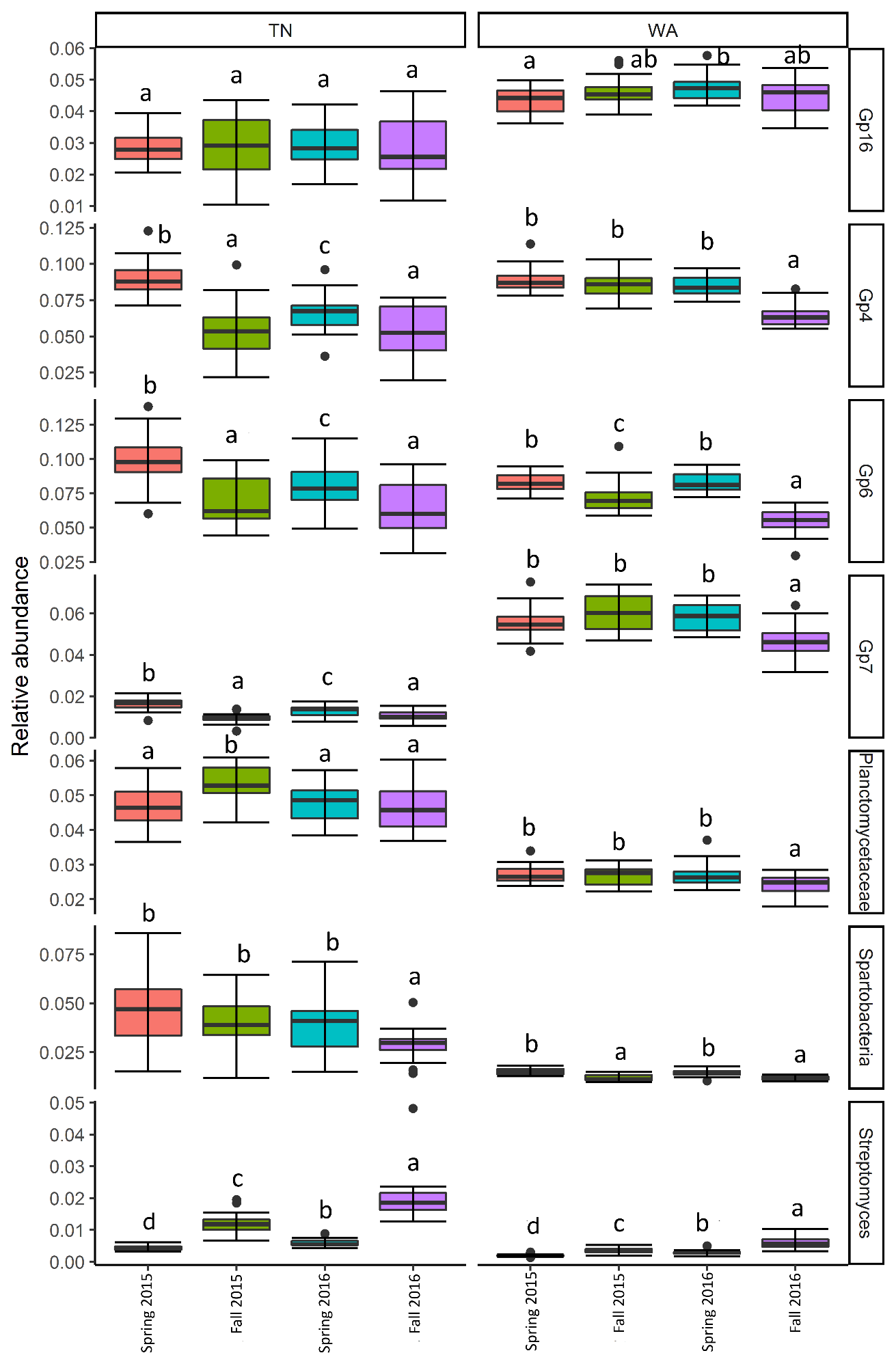


**Fig. S2: Percent relative abundance (averaged across treatments) of the most influential genera cumulatively responsible for about 60% of the seasonal variance between microbial communities**. Family and class level classification was used where classification at genus level was not possible. “Gp” indicates acidobacterial subgroups. Data from Spring 2015 show baseline condition, prior to mulch application. The lower and upper hinges of the boxplots correspond to the 25th and 75th percentiles and whiskers extend to the greatest value no further than 1.5 times the inter-quartile range. Data beyond the end of the whiskers are outliers and are plotted individually. Letters indicate post-hoc groupings determined by a pairwise Wilcoxon rank sum test using significance at α = 0.05.


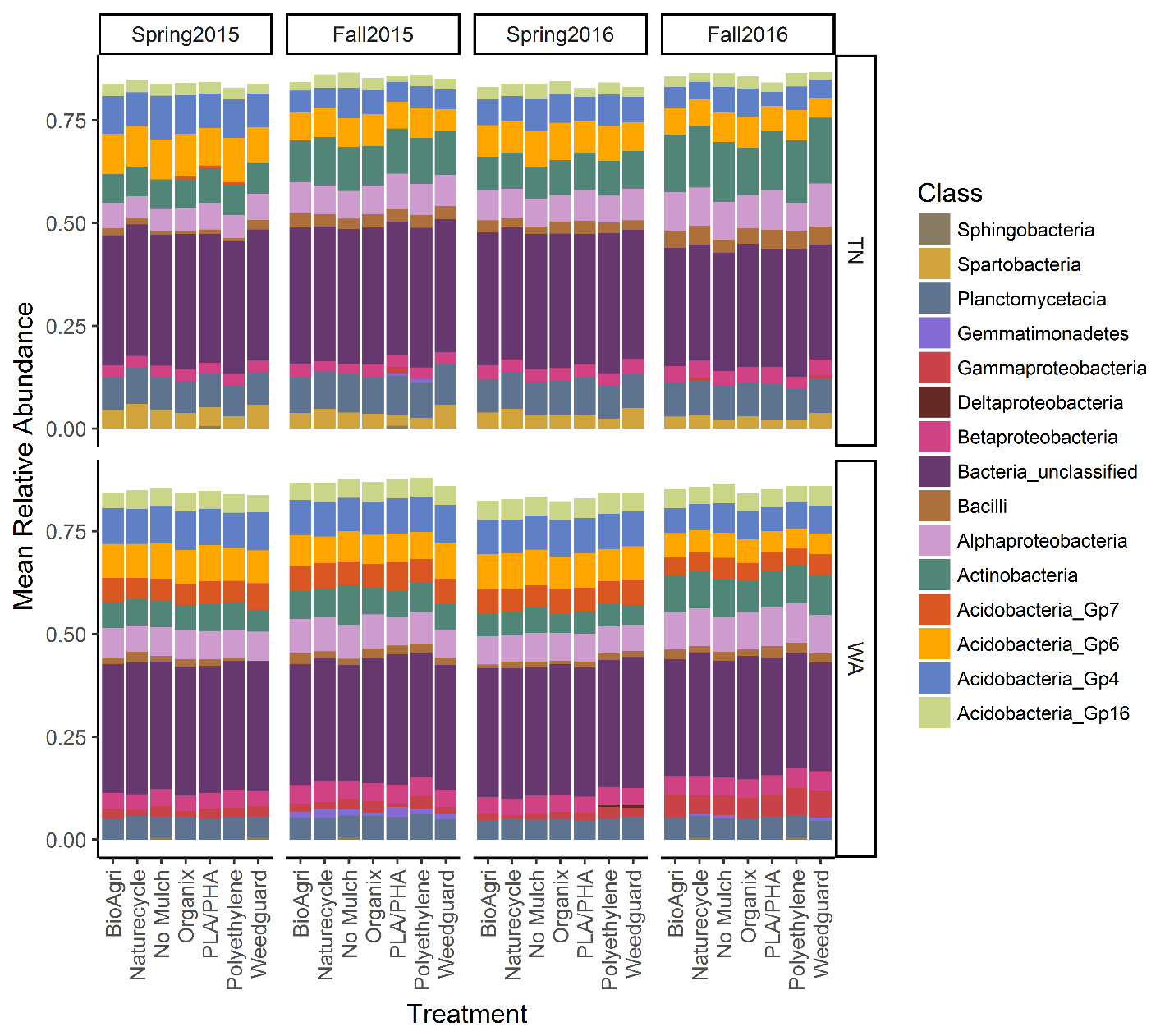


**Fig. S3: Stacked bar plots of depicting the abundant classes of bacteria in TN and WA for all mulch treatments.** Data from Spring 2015 show baseline condition, prior to mulch application.


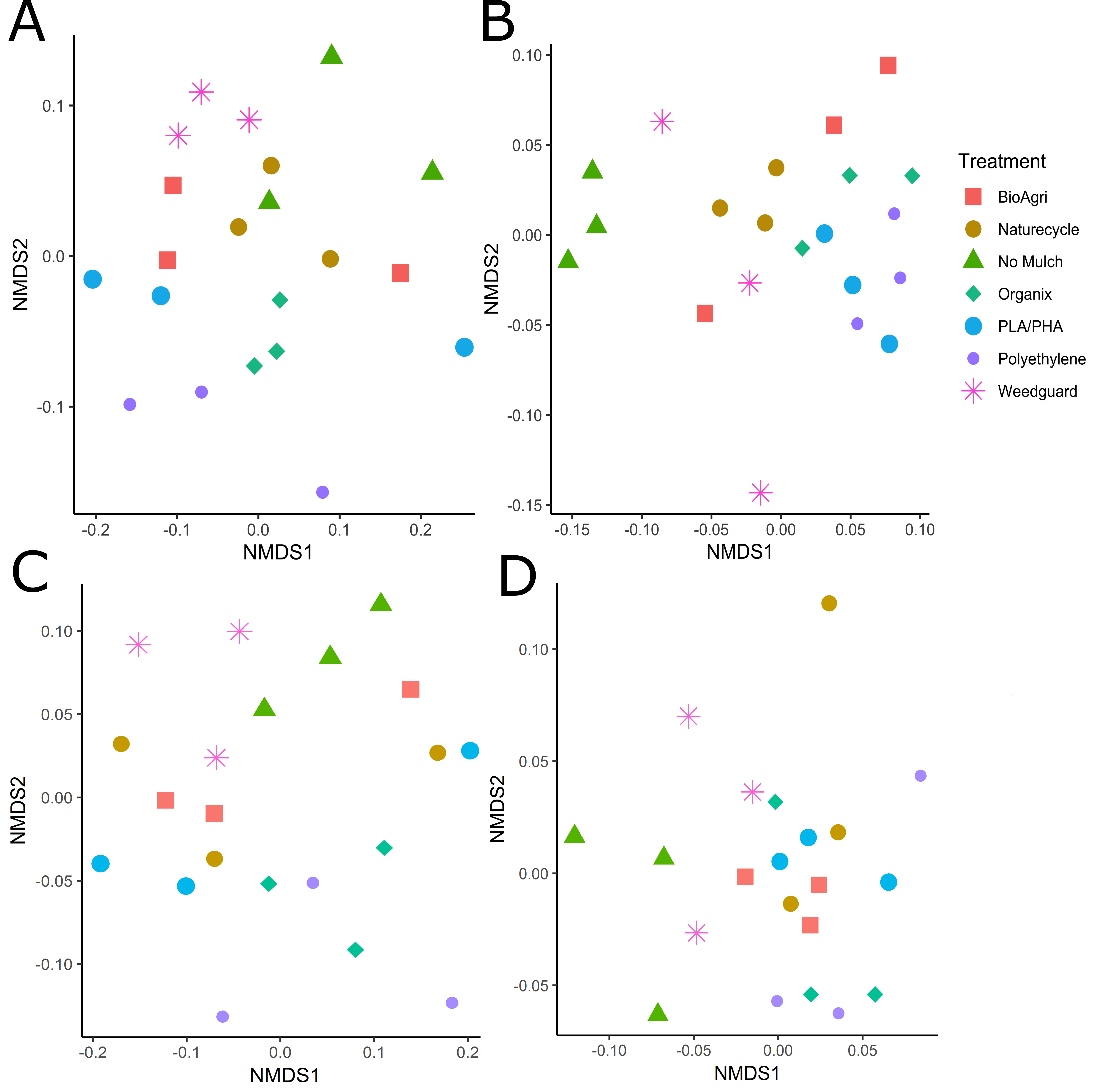


**Fig. S4: NMDS of soil bacterial community structures at the fall samplings.** A) 2015, TN (stress: 0.15); B) 2015, WA (stress: 0.16); C) 2016, TN (stress: 0.14); and D) 2016, WA (stress: 0.2). No significant difference between treatments were observed.

a)


b)

**Fig. S5: NMDS plot of soil extracellular enzyme activities from samples collected in Spring 2017 (final time point for enzyme rate measurements) for a) TN (p > 0.05) and b) WA (p > 0.05).**

**Reference**

Sintim HY, Bandopadhyay S, English ME, Bary AI, DeBruyn JM, Schaeffer SM, Miles CA, Reganold JP, Flury M. 2019. Impacts of biodegradable plastic mulches on soil health. Agriculture, Ecosystems & Environment 273:36-49.
